# Kinder und Jugendliche in der COVID-19-Pandemie – zur besonderen Betroffenheit einer vermeintlichen „low risk group“

**DOI:** 10.1007/s00103-021-03462-2

**Published:** 2021-12-10

**Authors:** Anke Spura, Nadine Reibling, Heidrun M. Thaiss, Freia De Bock

**Affiliations:** 1 Referat Q 4 Forschungskoordination, -kooperation, Wissenstransfer, Bundeszentrale für gesundheitliche Aufklärung (BZgA), Maarweg 149–161, 50825 Köln, Deutschland; 2 Leitung BZgA a.D., Bundeszentrale für gesundheitliche Aufklärung (BZgA), Maarweg 149–161, 50825 Köln, Deutschland; 3 Technische Universität München TUM, Georg-Brauchle Ring 60/62, 80992 München, Deutschland; 4 Abteilung Q Forschung, Zielgruppen, Lebenslagen, Bundeszentrale für gesundheitliche Aufklärung (BZgA), Maarweg 149-161, 50825 Köln, Deutschland

Zu Beginn der COVID-19-Pandemie galt es zunächst, die Gesundheit von alten Menschen und Menschen mit Vorerkrankungen, die ein hohes Risiko für einen schweren Verlauf einer COVID-19-Erkrankung trugen, zu schützen. Die Situation von Kindern und Jugendlichen stand nicht im Fokus der Maßnahmen zum Schutz vor einer SARS-CoV-2-Infektion – weder in der Politik noch in der Öffentlichkeit. Dass solche Maßnahmen zum Schutze der einen Gruppe aber auch Auswirkungen auf die Gesundheit anderer Gruppen haben können, wird über die letzten Monate immer deutlicher. Studiendaten der jüngsten Zeit (COPSY, DAK, COSMO u. a.) belegen deutlich, dass sowohl die psychischen als auch die physischen Folgen der nichtpharmakologischen Maßnahmen für Kinder und Jugendliche ein noch nicht abschätzbares Ausmaß erreicht haben.

Somit steht die Gesundheit von Kindern im Gegensatz zum Beginn der Pandemie nun deutlich im Vordergrund. Auch das Infektionsgeschehen selbst unterliegt einem Wandel. Nicht nur kann sich durch neue Mutationen das Risiko für symptomatische Erkrankungen und deren Folgen wie das multisystemische Entzündungssyndrom bei Kindern (PIMS) und Long-COVID bei Kindern ändern; auch die Bedeutung von Kindern und Jugendlichen für das allgemeine Infektionsgeschehen, im öffentlichen Raum und insbesondere in Schulen und Kindertagesstätten, hat sich zu einer wichtigen Forschungsfrage entwickelt. Die mit der Pandemie verbundenen Infektionsschutzmaßnahmen wie die Schließung von Schulen und Kitas, das Tragen von Masken, Kontaktverbote und Homeschooling haben den Alltag und das Leben von Kindern und Jugendlichen massiv verändert und ihre Chancen auf soziale und kulturelle Teilhabe beeinflusst. Inzwischen liegt eine Empfehlung zur Impfung mit einem zugelassenen mRNA-Impfstoff (Stand September 2021) für Kinder und Jugendliche ab 12 Jahren vor. Perspektivisch könnten COVID-19-Impfungen auch für Kinder unter 12 Jahren zugelassen und in eine nationale Impfstrategie integriert werden. Allerdings wird wie auch bei anderen Impfdiskursen die Kommunikation für diese Altersgruppe gesellschaftlich breit und sehr emotional geführt.

Internationale Daten zeigen, dass die Auswirkungen der pandemiebezogenen Maßnahmen auf die Gesundheit von Kindern und Jugendlichen auch weltweit eine immense Tragweite haben. Laut UNESCO [1] waren am bisherigen Höhepunkt der Schulschließungen im April 2020 für 1,4 Mrd. in Ausbildung befindliche Kinder, Jugendliche und Erwachsene die entsprechenden Einrichtungen geschlossen. Dabei zeigen sich deutliche Unterschiede zwischen den Ländern: Während die Schulen in Island nur 6 Wochen nicht geöffnet hatten, sind die Schulen in den USA und Indien seit über einem Jahr durchgehend geschlossen. Deutschland liegt mit einer Schließzeit von 30 Wochen im Mittelfeld. Auf Basis des Oxford COVID-19 Government Response Tracker [2] lässt sich sogar tagesgenau nachvollziehen, wie strikt und umfassend die Maßnahmen in den jeweiligen Ländern im gesamten Pandemieverlauf waren. Die interaktive Karte zeigt, dass wahrscheinlich der Großteil der Kinder und Jugendlichen dieser Welt von diesen Maßnahmen betroffen war. Darüber hinaus zeigt sich aber auch, dass das Ausmaß der Einschränkung von Bildungs- und Entwicklungschancen ungleich verteilt und nicht nur vom Infektionsgeschehen, sondern von weiteren Kriterien abhängig war, die die politischen Entscheidungen maßgeblich beeinflusst haben.

Damit sind die Folgen der Maßnahmen für die Gesundheit und Entwicklung von Kindern und Jugendlichen zu einer Frage geworden, mit der sich Forschung und Gesellschaft auch perspektivisch intensiv auseinandersetzen müssen. Dabei ist noch unklar, wie nachhaltig die Auswirkungen für Kinder und Jugendliche sein werden und Entwicklungs- und Lebensverläufe geprägt werden. Diese Fragen können aktuell nur bedingt beantwortet werden; eine langfristige Perspektive ist aber unabdingbar, da die sozialen und wirtschaftlichen Auswirkungen der COVID-19-Pandemie auf die Gesundheit von Kindern und Jugendlichen auch nach einem Abflachen des Infektionsgeschehens bereits absehbar sind. Sicher ist, dass insbesondere Kinder in vulnerablen Lebenslagen besonders betroffen sein werden. Familienpolitik und soziale Sicherungssysteme sind deshalb zentral, um negative Auswirkungen gerade auf diese Gruppe abzufedern [3].

Dass Effekte bevölkerungsbezogener Maßnahmen auf die Gesundheit vor allem jene Gruppen treffen, die bereits vorab belastet waren, wird dabei zu einer Herausforderung. Zudem stellen sich rechtliche und ethische Fragen, die sich auch in den Diskussionen um Kinderrechte als Grundrechte der Verfassung spiegeln. Neben sozialen und entwicklungsgefährdenden Folgen für Kinder und junge Menschen und damit assoziierten Kosten könnten Generationenverträge und -beziehungen belastet und damit indirekt die Stabilität der Gesellschaft gefährdet werden. All dies zeigt, dass es zwingend erforderlich ist, den Blick nicht nur auf die verschiedenen Bevölkerungsgruppen und ihre Vulnerabilität, sondern auch auf die Betroffenheit weit über das infektiologische Geschehen hinaus zu weiten. Hierauf ist dieses Heft u. a. ausgerichtet, wenngleich es damit ebenfalls relevante Themen eher ausspart wie Long-COVID oder Impfungen für Kinder. Hier sei auf die bisherigen Schwerpunkthefte des Bundesgesundheitsblattes aus der Reihe „COVID-19 und Public Health“, aber auch auf zukünftige Beiträge verwiesen.

Zu Beginn geben *Thamm et al.* eine Übersicht über die SARS-CoV-2-Seroprävalenz bei Kindern und Jugendlichen in Deutschland und zeigen damit die unmittelbare Belastungslage innerhalb des Infektionsgeschehens. *Kern et al.* gehen der Frage nach, welche Rolle Schul- und Kitakinder bei der Übertragung des SARS-CoV-2-Virus spielen.

Vor diesem epidemiologischen Hintergrund wird die psychosoziale Belastungslage ausführlicher thematisiert, der Kinder und Jugendliche sowie Eltern während dieser Pandemie ausgesetzt sind. Die verschiedenen Aspekte werden in den Beiträgen der Autorengruppen um *Rabe*, um *Ravens-Sieberer*, um *Döpfner* sowie um *Naumann* analysiert, ergänzt um den Beitrag von *Bantel et al.*, der die Effekte auf das kindliche Entwicklungsgeschehen basierend auf der Auswertung von Schuleingangsuntersuchungen untersucht.

Weitere mittelbare, in der Zukunft liegende Effekte sind nicht nur im Schulbereich durch ungleiche Entwicklungsstände erwartbar, sondern auch durch veränderte Inanspruchnahmen von präventiven Versorgungsleistungen wie Impfungen und Früherkennungsuntersuchungen. In dieses Thema geben *Mangiapane et al.* auf der Grundlage von Routinedaten einen Einblick. *Heudorf et al.* zeigen aus der Perspektive des Öffentlichen Gesundheitsdienstes die Effekte im Zusammenhang mit kindlichen Entwicklungsmöglichkeiten auf.

Die Lebenslagen und Lebenswelten von Kindern sind in den folgenden Beiträgen im Zentrum. *Birmili et al.* widmen sich den Lüftungskonzepten in Schulen und *Loss et al.* den Maßnahmen in den Kitas. *Freiberg et al.* setzen sich in diesem Zusammenhang mit dem Einfluss des Tragens von Masken auf die psychosoziale Entwicklung von Kindern und Jugendlichen auseinander. Unterstützung von Kindern und Familien in belastenden Lebenslagen leisten die Frühen Hilfen. *Renner et al.* schildern die Herausforderungen und Chancen der „Hilfen aus der Distanz“ unter den Bedingungen des Lockdowns.
